# Morphological characterization and RNA sequencing reveal adaptive strategies of *Coix lacryma*-*jobi* L. under waterlogging stress during the jointing stage

**DOI:** 10.7717/peerj.20731

**Published:** 2026-02-05

**Authors:** Jing Yao, Zhiqing Gong, Weijie Tang, Mei Yuan, Yunyan He, Yantao Liang, Weizhong Li, Ke Zhong, Dandan Wang, Peilong He

**Affiliations:** School of Pharmacy, Guizhou University of Traditional Chinese Medicine, Guiyang, Huaxi, China

**Keywords:** *Coix lacryma-jobi* L., Waterlogging, RNA-sequencing, Photosynthesis, Carbohydrate metabolism

## Abstract

**Background:**

*Coix lacryma*-*jobi* L. is a vital medicinal and dual-purpose crop in Guizhou, requiring optimal cultivation conditions to preserve its therapeutic properties. Waterlogging stress significantly reduces its productivity, yet the underlying molecular mechanisms remain poorly understood.

**Methods:**

We investigated the species’ adaptive responses using controlled waterlogging experiments on potted plants. Morphological alterations and key growth parameters (culm diameter, plant height, dry biomass) were quantified. We performed transcriptomic profiling by RNA-Seq to identify differentially expressed genes (DEGs). To elucidate the functional implications of these DEGs, we conducted systematic enrichment analyses using the clusters of orthologous groups (COG), Gene Ontology (GO), and Kyoto Encyclopedia of Genes and Genomes (KEGG) databases. Expression patterns of candidate genes were validated using Quantitative Real-Time Reverse Transcription Polymerase Chain Reaction (qRT-PCR).

**Results:**

Flooded plants exhibited significant morphological changes, with key growth parameters decreasing by 14.4–21.8%. A pronounced adaptive response was a 76.6% increase in adventitious root formation. RNA-Seq revealed 207 DEGs (133 upregulated, 74 downregulated). Enrichment analyses indicated these DEGs were predominantly involved in photosynthetic processes, carbohydrate dynamics, and signaling pathways. qRT-PCR confirmed consistent expression patterns for six candidate genes associated with photosynthesis and carbohydrate metabolism. These findings demonstrate that *Coix lacryma*-*jobi* L. activates coordinated molecular responses, including photosynthetic efficiency adjustment and metabolic pathway remodeling, to enhance waterlogging tolerance. This work identifies critical genetic components governing waterlogging adaptation, providing molecular markers for developing stress-tolerant cultivars through targeted breeding.

## Introduction

As a globally prevalent form of abiotic stress, waterlogging poses substantial threats to agricultural sustainability, currently affecting approximately 16% of arable lands (Zhou et al., 2020). This environmental challenge is projected to intensify under climate change (Donat et al., 2017), necessitating urgent investigations into plant adaptation mechanisms. The hypoxic microenvironment induced by waterlogging triggers a cascade of physiological disturbances: soil oxygen depletion forces root systems to initiate anaerobic respiration, generating cytotoxic metabolites such as ethanol and acetaldehyde (Kornyeyev et al., 2001). Concurrently, photosynthetic impairment arises from CO_2_ limitation due to stomatal closure and chloroplast ultrastructure damage, exacerbated by reduced irradiance during precipitation events (Du et al., 2022). Nutrient acquisition deficits further compound these stresses as a result of soil nutrient leaching, mycorrhizal symbiont mortality, and impaired root functionality (Colmer & Voesenek, 2009).

Morphological plasticity constitutes a primary adaptive strategy under hypoxic conditions. The development of adventitious roots (ARs) represents a critical survival mechanism, enabling waterlogged plants to maintain resource acquisition through functional root replacement (Qi et al., 2020). AR morphogenesis is regulated through synergistic interactions among the ethylene, auxin, nitric oxide, and hydrogen peroxide signaling pathways (Liao et al., 2015; Liao et al., 2012; Xu et al., 2017). Comparative analyses reveal interspecies variation in aerenchyma formation strategies. *Oryza sativa* exhibits constitutive aerenchyma development with adaptive plasticity under oxygen deprivation, whereas *Hordeum vulgare* requires hypoxic induction for limited aerenchyma production (Luan et al., 2018).

At the molecular level, plants activate sophisticated gas-sensing mechanisms and reprogram their metabolism to cope with hypoxic stress. Changes in the gaseous environment—such as ethylene and CO_2_ accumulation alongside O_2_ depletion—trigger signaling cascades through key secondary messengers. These include fluctuations in carbohydrate status, shifts in cytoplasmic pH, calcium oscillations, and reactive oxygen species (ROS) bursts (Sasidharan et al., 2017). Ethylene and hypoxia signaling interact to regulate critical adaptive responses (Voesenek, Sasidharan & Weber, 2013). Metabolically, plants rapidly induce anaerobic respiration pathways. Among these, pyruvate decarboxylase (PDC)-driven ethanol fermentation is essential for regenerating NAD^+^ and maintaining ATP levels (Geigenberger, 2003; Xu et al., 2017). Additionally, energy redistribution is facilitated by the dynamic regulation of carbohydrate metabolism enzymes, particularly sucrose synthase and invertase (Jie et al., 2016).

*Coix lacryma*-*jobi* is an orphan crop with dual uses for medicinal and food purposes, particularly in regions such as Guizhou, China. Beyond its well-known role as a source of polysaccharides for the food industry, modern pharmacological studies have revealed a broader spectrum of bioactivities, including immunomodulatory (Lee et al., 2024), antitumor activity (Chiang et al., 2022), hypoglycemic, and lipid-regulating effects (Li et al., 2024). Additional reported functions include melanogenesis inhibition (Amen et al., 2017; Huey-Chun et al., 2014) and anti-inflammatory and analgesic properties (Sui & Xu, 2022; Yang et al., 2013). The hard, stony seed coat of *Coix lacryma*-*jobi* poses a challenge for consumption and processing. Therefore, mechanical dehulling—typically performed using abrasive milling or roller shellers—is commonly employed to produce edible groats, a process analogous to that used for other small grains (Qin et al., 2021).

Ecologically, as a member of the Poaceae family often found in humid, rain-fed habitats, it is frequently exposed to periodic waterlogging, suggesting the presence of inherent adaptive mechanisms. Its close phylogenetic relationship with major cereals such as maize and sorghum also positions it as a valuable model for studying waterlogging responses in gramineous plants (Zhao, 2000).

Research on abiotic stress in *Coix lacryma*-*jobi* has mainly focused on drought (Zhang et al., 2023), low temperatures (Huang et al., 2019), and heavy metals (Yu et al., 2022; Zhao et al., 2024), *etc.*, with relatively few studies on waterlogging stress. Current studies on waterlogging stress in *Coix lacryma*-*jobi* mainly focus on its effects on physiological indexes, and the molecular mechanisms of this species’ response to waterlogging stress require further investigation. This study aims to address critical knowledge gaps in the stress responses of *Coix lacryma*-*jobi* by (1) systematically evaluating the morpho-physiological adaptations of the Guizhou-endemic Xiaobaike cultivar under controlled waterlogging conditions; (2) elucidating the molecular mechanisms underlying waterlogging tolerance through RNA-Seq analysis, with particular focus on photosynthetic regulation, carbohydrate metabolism, and signaling pathways; and (3) identifying novel genetic elements that are responsive to hypoxic stress. By integrating phenotypic observations with RNA-Seq analysis comparing severe waterlogging treatments to controls, this work seeks to: (i) quantify stress-induced biomass reallocation patterns, (ii) characterize differentially expressed genes governing key adaptive processes, and (iii) establish a catalog of 1361 newly annotated genes as potential molecular markers. The findings provide the first comprehensive framework for understanding waterlogging adaptation strategies in *Coix lacryma*-*jobi*, offering both a theoretical foundation and practical genetic resources for developing waterlogging-resistant cultivars through molecular breeding approaches.

## Materials & Methods

### Plant materials and growth conditions

In this study,we utilized *Coix lacryma*-*jobi* ‘Xiaobaike’ (Poaceae), a medicinal cultivar originally obtained from the Germplasm Repository of Guizhou Herbal Resources. *Coix lacryma*-*jobi* is a cross-pollinated species featuring a high degree of genetic heterogeneity among individuals. To minimize the impact of genetic variation in this study, ‘Xiaobaike’ specimens were sourced from a certified supplier and grown under uniform conditions to reduce pre-experimental variation. Cultivation occurred in a climate-controlled greenhouse at the same institution, where the plants were maintained under standardized phytotron conditions: a 16/8 h photoperiod (light/dark), an ambient temperature maintained at 20–25 °C, and a relative humidity stabilized at 70 ± 5% throughout the experimental period. To mitigate the effects of spatial heterogeneity in environmental conditions, the plant pots were periodically rotated throughout the cultivation period.

### Germination conditions and the experimental design of the potted plants

*Coix lacryma*-*jobi* cv. Xiaobaike seeds of uniform size were subjected to thermal pretreatment in aerated water (50–55 °C, 30 min) to eliminate defective specimens. Germination was carried out in controlled climate chambers (25 °C, RH 70%), followed by transplantation to nursery substrates. The jointing stage was considered to be when the main culm exhibited a palpable internode elongation of approximately 1–2 cm above the surface of the soil, synchronized with the emergence of the flag leaf sheath (the penultimate leaf), which aligns with the critical developmental transition from vegetative to reproductive growth as described in cereal phenology (Zhuang & Lijuan, 2006). Twenty-four vigorous seedlings at the three-leaf stage were randomly allocated into four experimental cohorts (*n* = 6 per group): non-stressed control (CK) and three waterlogging intensities-light water logging (LW, water maintained at soil surface level), moderate waterlogging (MW, +1 cm above soil surface), and severe waterlogging (SW, +2.5 cm above soil surface). The waterlogging treatments followed established protocols with modifications (Song et al., 2025) and were imposed during the critical stem elongation phase (46–65 days post-transplantation), a key developmental period as described in previous studies (Zhao, 2000). Hydric conditions were strictly monitored through daily volumetric supplementation to maintain predefined water table elevations, with the control plants receiving standard irrigation matching evapotranspiration demands.

### Assessment of morphological responses to waterlogging stress

Plant architecture analysis was conducted after 19 days of controlled waterlogging treatment. Plant height was determined by measuring the vertical distance from the soil surface to the panicle tip using standardized procedures (Lin et al., 2009). Three representative leaves (flag leaf, penultimate leaf, and third apical leaf) were selected for dimensional analysis, with the maximum lamina length and maximum midrib width recorded. Culm diameter was measured at the central internode midpoint using digital calipers (Mitutoyo 500-196, ±0.01 mm precision).

Nodal development parameters were evaluated post-treatment. Primary tillers were analyzed for the total internode number and individual segment length through serial measurements along the main culm. To maintain measurement consistency, leaf dimensions were acquired at the midpoint between the ligule and leaf tip, while culm diameter measurements followed identical positional criteria as the baseline assessments.

Dry matter content determination followed modified protocols from an established methodology (Wang et al., 2020). Experimental and control plants were carefully rinsed to remove soil particles, surface moisture was blotted with absorbent paper, and the fresh weight was recorded. The samples were then dehydrated in a convection oven at 70 °C until a constant mass was achieved. The dry matter percentage was computed using the following formula: Dry matter (%) = (Dry weight/Fresh weight) × 100.

### RNA-Seq analysis

The RNA-Seq analysis protocol was adapted from Pertea et al. (2015) with modifications. This study utilized three biological replicates per experimental group (control and the severe waterlogging treatment), harvested at the jointing growth stage. No technical replicates were performed. Based on an assumed effect size of 2 (log2 fold change), a sequencing depth of 24.12 million reads per sample, and an adjusted significance threshold (FDR) of 0.01, a power analysis using RNASeqPower indicated a statistical power of 0.72 for detecting differentially expressed genes under this experimental design.

Total RNA was extracted from root after 19 days of waterlogging treatment using TRlzol Reagent (Life Technologies, Carlsbad, CA, USA) and assessed for quality using the NanoDrop 2000 (Thermo Fisher Scientifi, Waltham, MA, USA) for concentration/purity and the RNA Nano 6000 Assay Kit on the Agilent 2100 Bioanalyzer for integrity. Libraries were prepared from 1 µg of high-quality RNA per sample using the Hieff NGS Ultima Dual-mode mRNA Library Prep Kit (Yeasen Biotech, Shanghai) with manufacturer-specified barcoding. Paired-end sequencing (150 bp reads) was performed on an Illumina NovaSeq platform following standard protocols. Reads were aligned to the reference genome (accession: GWHAAYR00000000) using HISAT2 v2.0.4, achieving an average mapping rate of 90% across all samples.

Functionally annotated genes were assigned based on alignments to multiple databases, including NCBI non-redundant protein (Nr), Protein family (Pfam), Clusters of Orthologous Groups (KOG/COG), Swiss-Prot, KEGG Orthology (KO), and Gene Ontology (GO). Differential gene expression analysis was conducted using DESeq2, applying a negative binomial generalized linear model. Genes were considered significantly differentially expressed if they met an adjusted *P*-value (Benjamini–Hochberg FDR) threshold of <0.01 and exhibited a minimum 2-fold change in expression.

The raw RNA-Seq data generated in this study have been deposited in the NCBI Sequence Read Archive (SRA) and are publicly accessible under the accession number PRJNA1304239.

### Gene expression profiling *via* quantitative reverse transcription PCR

The qRT-PCR protocol was adapted with modifications from an established methodology (Zhang et al., 2024). Six target genes exhibiting |log2FC| values >5 and functional relevance to photosynthetic pathways and carbohydrate metabolism were selected for validation (Table S1). Root tissue samples from the experimental and control groups were homogenized in RNAprep Pure Plant Kit (Tiangen Biotech (Beijing) Co., Ltd, Beijing, China) for total RNA extraction. RNA purity and structural integrity were evaluated spectrophotometrically (NanoDrop 2000, Thermo Fisher Scientific, Waltham, MA, USA) by determining A260/A280 and A260/230 ratios, supplemented with electrophoretic verification using 1% agarose gels. Residual genomic DNA was removed using StarScript Pro All-in-one RT Mix with integrated gDNA elimination (GenStar), followed by first-strand cDNA synthesis.

Amplification reactions were performed in triplicate using a Bio-Rad CFX Connect Real-Time PCR platform (Bio-Rad, Hercules, CA, USA) with 2×RealStar Fast SYBR qPCR Master Mix (GenStar). Primer pairs specific to the target sequences and the 18S rRNA reference gene were designed, as detailed in Table S1. Thermal cycling parameters followed the manufacturer specifications. To ensure experimental reliability, three biological replicates, each containing three technical repeats, were analyzed. Transcript abundance quantification was conducted using the comparative threshold cycle (2^−ΔΔCt^) method for relative expression analysis.

### Statistical analysis

Data collection and management were conducted using WPS Office v12 (WPS Cloud, Beijing, China). Image post-processing and enhancement were conducted using Adobe Photoshop 2024 (Adobe Systems Inc., San Jose, CA, USA). For quantitative analyses, statistical computations and data visualization were performed in GraphPad Prism v9.5 (GraphPad Software, San Diego, CA, USA), applying one-way ANOVA with Tukey’s *post-hoc* test for multi-group comparisons. Three independent experimental replicates were conducted to verify methodological reliability. The quantitative results were presented as mean ± SEM, with the corresponding *p*-values indicated for statistical significance.

## Results

### Effects of waterlogging stress on morphological development in *Coix lacryma*-*jobi* at the jointing stage

#### Plant height and culm development responses to waterlogging stress in *Coix lacryma*-*jobi*

**Figure 1:**
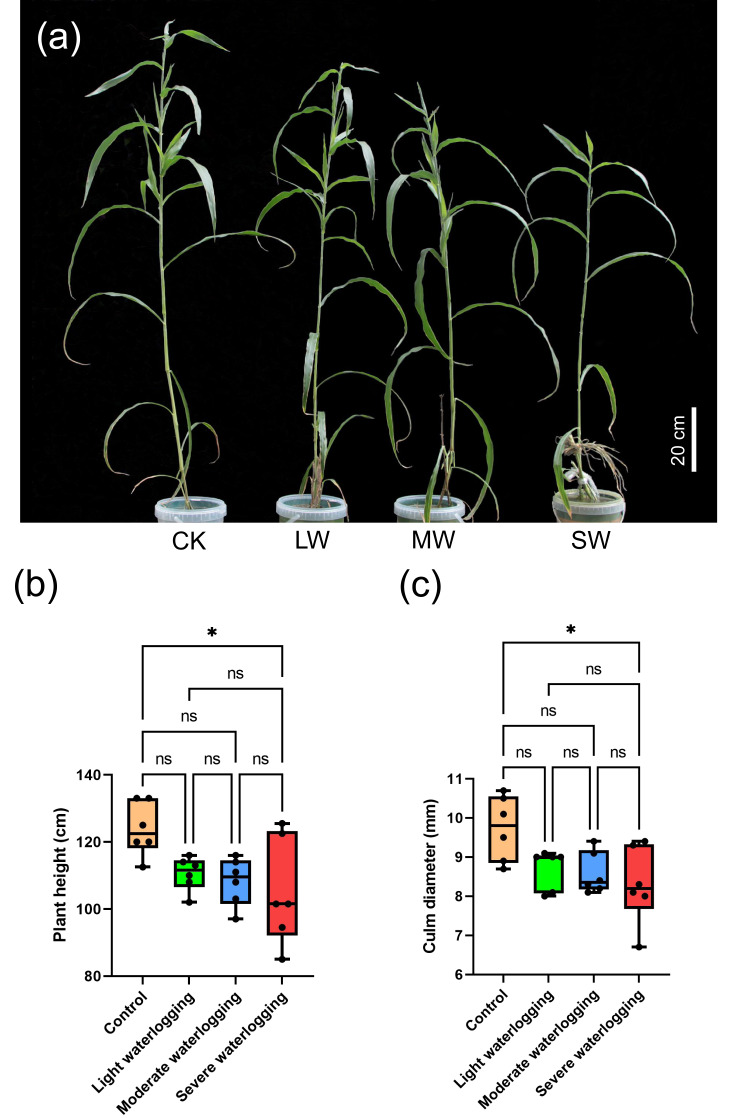
Effects of waterlogging stress on plant height and culm diameter in *Coix lacryma*-*jobi* during the jointing stage. (A) Photograph of representative plants from each treatment group indicating the phenotypes of waterlogging-treated and control plants. CK, control; LW, light waterlogging; MW, moderate waterlogging; and SW, severe waterlogging. (B) Plant height under different waterlogging durations. (C) Culm diameter of waterlogging-treated and control plants. Asterisks indicate signiffcant differences from control: *, *p* < 0.05 (one-way ANOVA with Tukey’s *post-hoc* test).

The differential responses of *Coix lacryma*-*jobi* to varying hydric stress intensities manifested as distinct morphological adaptations (Fig. 1). Although severe waterlogging induced significant reductions in vertical growth, with plant height decreasing by 9.8% relative to non-stressed controls, plants subjected to light and moderate treatments maintained comparable vertical development profiles (*p* > 0.05; Figs. 1A, 1B). This intensity-dependent pattern was replicated in culm diameter measurements, where the severe treatment group exhibited a 6.7% reduction in culm dimensions compared to the control plants (*p* < 0.05), while no statistically discernible variations emerged among light/moderate treatments and control specimens (*p* > 0.05, ANOVA with Tukey’s *post-hoc*; Fig. 1C). Notably, inter-group comparative analyses confirmed the absence of significant morphometric differentiation between adjacent stress levels. These pronounced morphological responses to under waterlogging stress highlight the species’ differential tolerance thresholds, where different flooding depths seem to be critical for triggering growth inhibition.

### Internode elongation patterns under waterlogging stress at the jointing stage

**Figure 2:**
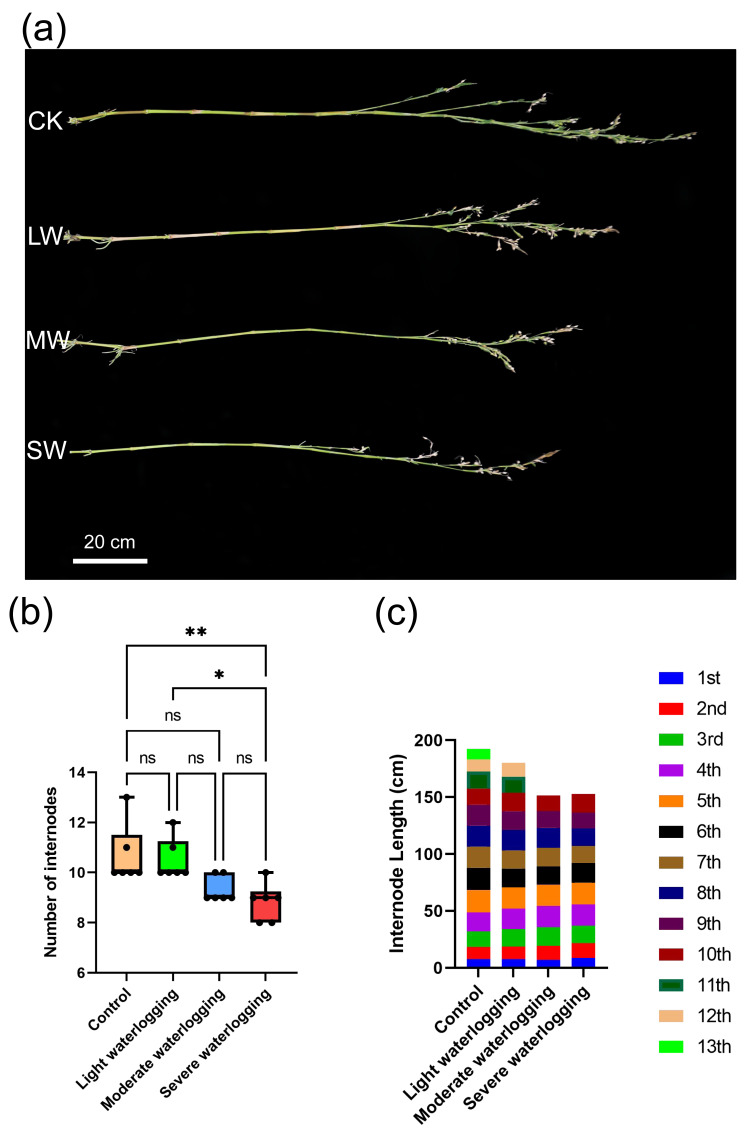
Effects of waterlogging on internode growth in *Coix lacryma*-*jobi* during the jointing stage. (A) Photograph of representative plants from each treatment group indicating the phenotypes of internodes under different treatments. CK, control; LW, light waterlogging; MW, moderate waterlogging; and SW, severe waterlogging. (B) Internode number per plant. (C) Internode length. Asterisks indicate significant differences from control: *, *p* < 0.05; **, *p* < 0.01 (one-way ANOVA with Tukey’s *post-hoc* test).

Waterlogging stress induced profound morphometric alterations in the nodal architecture of *Coix lacryma*-*jobi*. Quantitative analysis revealed striking variations in nodal segmentation patterns across experimental cohorts, with plants that received the severe waterlogging treatment displaying pronounced reductions in total internode counts relative to the non-stressed controls (*p* < 0.01, one-way ANOVA with Tukey’s HSD *post hoc* test). Notably, this treatment cohort also exhibited statistically distinguishable segmentation patterns compared to their moderate-stress counterparts (*p* < 0.05), while inter-group comparisons among other experimental conditions failed to reach statistical significance (Figs. 2A, 2B).

Longitudinal profiling of individual internodes uncovered stress-responsive elongation patterns, which were particularly evident in apical developmental zones. While most internodes maintained consistent dimensions across treatments, the seventh and ninth sub-apical internodes manifested significant length reductions under severe waterlogging conditions (19.7% and 23.8% decreases *vs.* controls, respectively; *p* < 0.05) as detailed in Fig. 2C and Table S2. This spatial specificity suggests that nodal meristems have differential sensitivities along the developmental gradient.

These findings collectively elucidate the dual regulatory effects of waterlogging stress on internodal morphogenesis: suppressing both meristematic initiation frequency and anisotropic cell expansion processes. The dose-dependent phenotypic responses, particularly the apical dominance in elongation inhibition, provide critical insights into hypoxia-mediated developmental modulation in monocot stem systems.

### Root architectural plasticity in *Coix lacryma*-*jobi* subjected to waterlogging during the jointing stage

**Figure 3:**
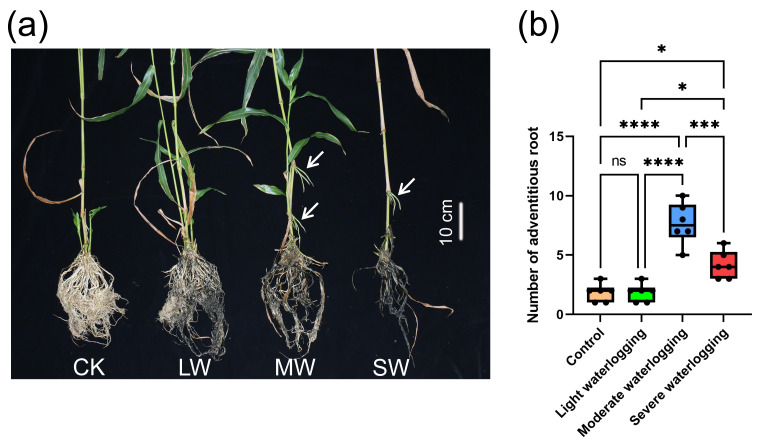
Effects of waterlogging on root development in *Coix lacryma*-*jobi* during the jointing stage. (A) Photograph of representative plants from each treatment group indicating the phenotypic comparison of adventitious roots between control and waterlogging treatment groups. CK, control; LW, light waterlogging; MW, moderate waterlogging; and SW, severe waterlogging. Arrowheads indicate adventitious roots. (B) Quantitative analysis of adventitious root number under different waterlogging conditions. Asterisks indicate significant differences from control: *, *p* < 0.05; ***, *p* < 0.001; ****, *p* < 0.0001 (one-way ANOVA with Tukey’s *post-hoc* test).

Waterlogging stress exerted substantial inhibitory effects on the architectural development of *Coix lacryma* root systems. Under controlled conditions, the primary roots maintained structural integrity and exhibited characteristic white pigmentation (Fig. 3A). Progressive darkening and tissue maceration became evident with escalating waterlogging intensity, accompanied by the gradual deterioration of root biomass (Fig. 3A).

Remarkably, stem-borne adventitious roots emerged as a prominent adaptive response, particularly under the moderate (MW) and severe (SW) waterlogging treatments. The MW group demonstrated maximal root induction (7.7 ± 1.7 roots/plant), surpassing both the control (1.8 ± 0.7, *p* < 0.0001) and light waterlogging (LW: 1.8 ± 0.7, *p* < 0.0001) groups. Although the SW plants showed reduced root formation (4.2 ± 1.1) compared to the MW plants (*p* < 0.0001), their root numbers still significantly exceeded control levels (*p* < 0.05). No statistical distinction emerged between the LW and control groups (*p* > 0.05) (Fig. 3B).

This non-linear response pattern suggests a threshold-dependent activation of adventitious root primordia. The peak induction at moderate stress implies an optimal balance between hypoxia signaling and metabolic competence, while severe stress likely imposes physiological constraints on root organogenesis.

### Waterlogging-driven alterations in dry matter partitioning at the jointing stage

**Figure 4:**
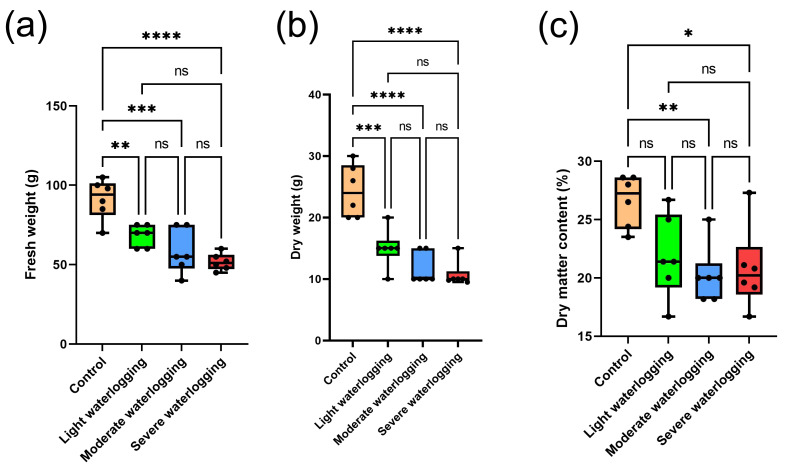
Effects of waterlogging treatments on plant biomass accumulation. (A) Fresh weight of plants under different waterlogging durations. (B) Dry weight of plants under different waterlogging durations. (C) Dry matter accumulation patterns across treatment groups. Asterisks indicate significant differences from control: *, *p* < 0.05; **, *p* < 0.01; ***, *p* < 0.001; ****, *p* < 0.0001 (one- way ANOVA with Tukey’s *post-hoc* test).

The dose-dependent effects of hypoxic stress on biomass partitioning in *Coix lacryma*-*jobi* were systematically quantified through triaxial growth parameters. As shown in Fig. 4A, fresh biomass accumulation was progressively reduced along the stress gradient, with plants subjected to the light (*p* < 0.01), moderate (*p* < 0.001), and severe (*p* < 0.0001) water-saturated treatments exhibiting 25.2%, 36.1%, and 43.4% reductions, respectively, compared to the normoxic controls. Notably, inter-group comparisons among the stressed cohorts did not reach statistical significance (*p* > 0.05), suggesting threshold saturation in stress response mechanisms.

Dry biomass dynamics paralleled the trends observed in fresh weight but with amplified statistical differentiation (Fig. 4B). The quantile regression revealed 38.4% (*p* < 0.001), 52.1% (*p* < 0.0001), and 56.0% (*p* < 0.0001) decrements under incremental hypoxia, demonstrating more pronounced metabolic disruption in carbon fixation processes. This dichotomy between fresh and dry weight responses highlights differential regulation in water uptake *versus* photosynthetic assimilation under oxygen deprivation.

The dry matter content (DMC) displayed nonlinear modulation across treatment groups (Fig. 4C). Moderate and severe hypoxia induced 24.0% (*p* < 0.01) and 21.9% (*p* < 0.05) reductions, respectively. This bimodal response implies the activation of compensatory mechanisms activation during early stress stages, followed by metabolic collapse under prolonged inundation, providing critical insights into plant adaptive strategies under edaphic hypoxia.

### RNA-Seq analysis of *Coix lacryma*-*jobi* under severe waterlogging *vs.* control conditions at the jointing stage

#### Functional categorization of waterlogging-responsive genes through COG analysis

**Figure 5:**
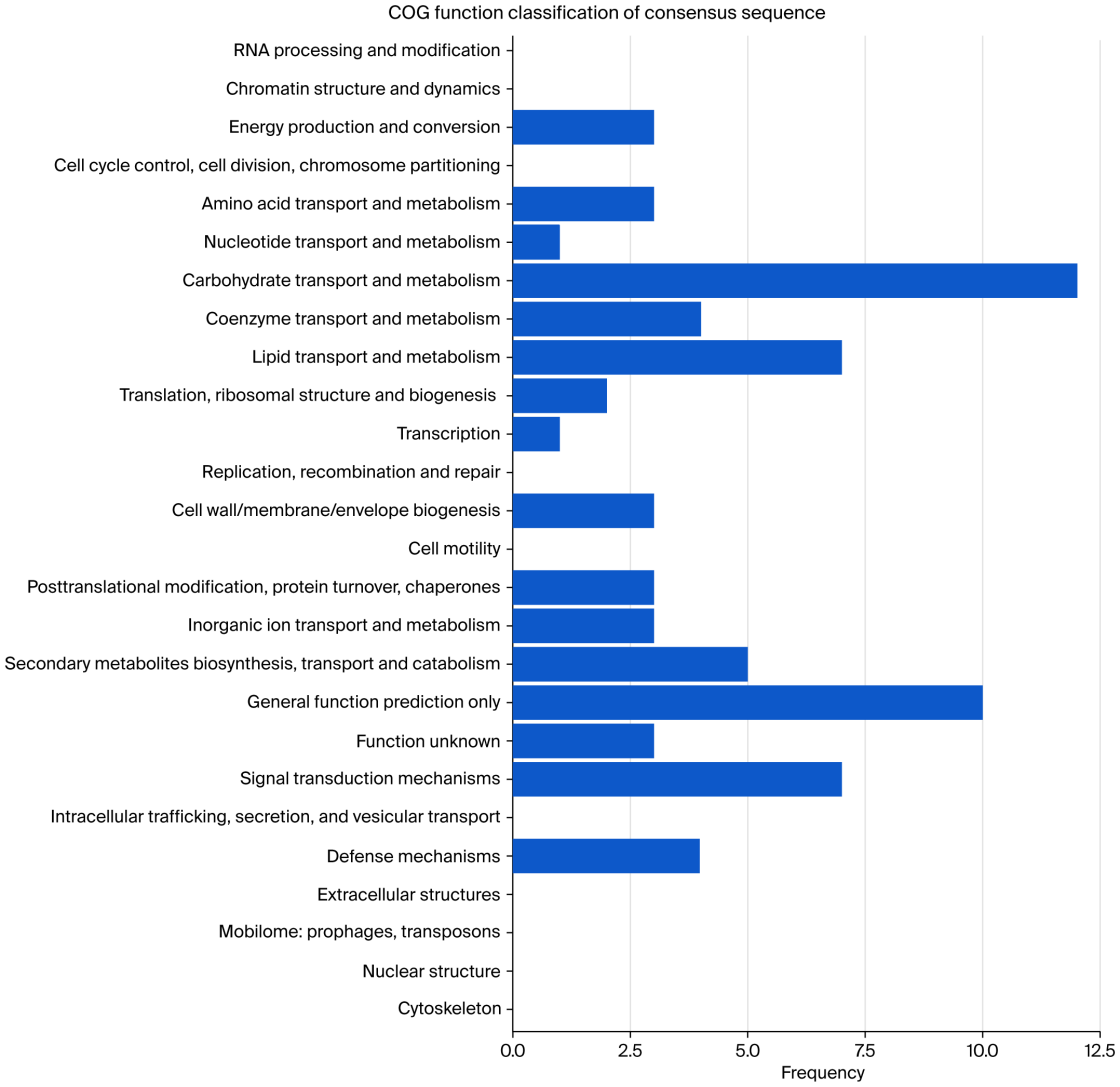
COG functional classification of differentially expressed genes (DEGs) in *Coix lacryma*-*jobi* under 19-day waterlogging treatment between control and waterlogging treatment groups.

RNA-Seq analysis revealed 207 significantly dysregulated genes (|log2FC| ≥1, FDR < 0.05) in waterlogging-stressed plants compared to untreated controls, comprising 74 induced and 133 suppressed transcripts (Table S3). Systematic COG functional categorization demonstrated statistically significant enrichment (Fisher’s exact test, *p* < 0.01) in six evolutionarily conserved modules critical for stress adaptation:

(i) Carbohydrate dynamics (transport and metabolic regulation), which are crucial for osmotic adjustment and energy homeostasis under hypoxia;

(ii) Lipid metabolic reprogramming, which is associated with membrane remodeling and signaling transduction;

(iii) Signal perception and amplification mechanisms, particularly receptor kinase-mediated cascades;

(iv) Biosynthesis and translocation of secondary metabolites, including phenolic compounds with antioxidant properties;

(v) Coenzyme-dependent metabolic networks regulating redox balance;

(vi) Pathogen defense systems, suggesting crosstalk between abiotic and biotic stress responses (Fig. 5, Table S4).

### Gene Ontology enrichment analysis of differentially expressed genes

Gene Ontology (GO) enrichment analysis demonstrated significant associations of differentially expressed genes with three principal ontological domains. Within biological processes, the predominant enriched terms encompassed metabolic processes, cellular processes, biological regulation, and responses to stimuli. Specifically, significant overrepresentation was identified in photosynthesis-related pathways (GO:0015979), protein–chromophore linkage (GO:0018298), methylation processes (GO:0032259), light response mechanisms (GO:0009416), and photosystem I light-harvesting functions (GO:0009768).

Analysis of cellular components revealed prominent enrichment in fundamental cellular architecture, including cellular anatomical entities (GO:0110165), intracellular structures (GO:0044424), and protein-containing complexes (GO:0032991). Distinctive subcellular localization patterns were observed for the chloroplast thylakoid membranes (GO:0009535), chloroplast stroma (GO:0009570), photosystem I (GO:0009522), photosystem II (GO:0009523), and chloroplast envelope (GO:0009941).

**Figure 6:**
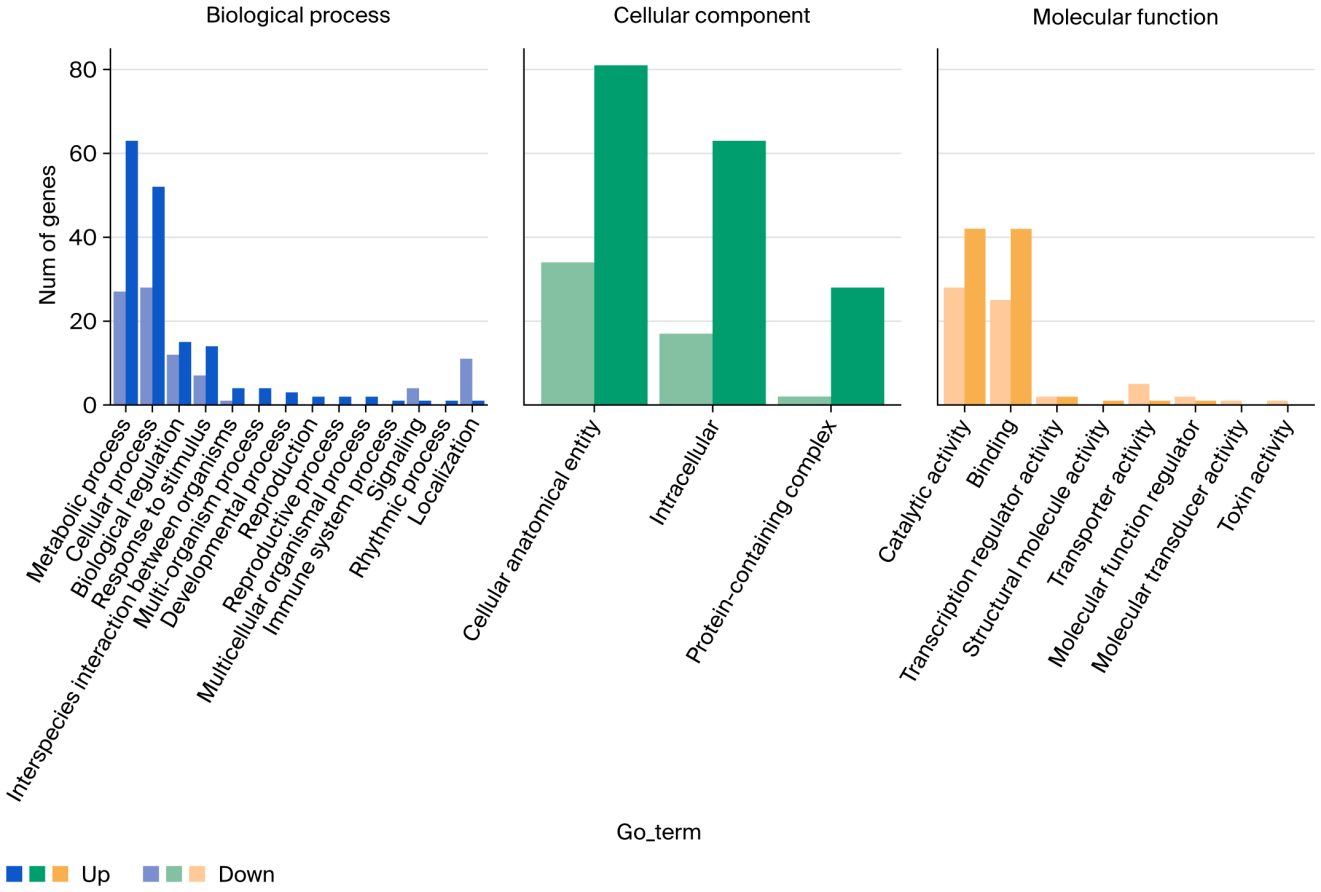
Bar plot of GO classification (biological process, cellular component, and molecular function) for DEGs.

Molecular function categorization highlighted two predominant activities: catalytic functions (GO:0003824) and molecular binding interactions (GO:0005488). Detailed analysis detected substantial enrichment in the chlorophyll binding capacity (GO:0016168), methyltransferase activity (GO:0008168), carbohydrate binding (GO:0030246), and transferase functions (GO:0016740), with complete statistical details presented in Fig. 6 and Figs. S1–S3 and Table S5.

### Kyoto Encyclopedia of Genes and Genomes pathway enrichment analysis

**Figure 7:**
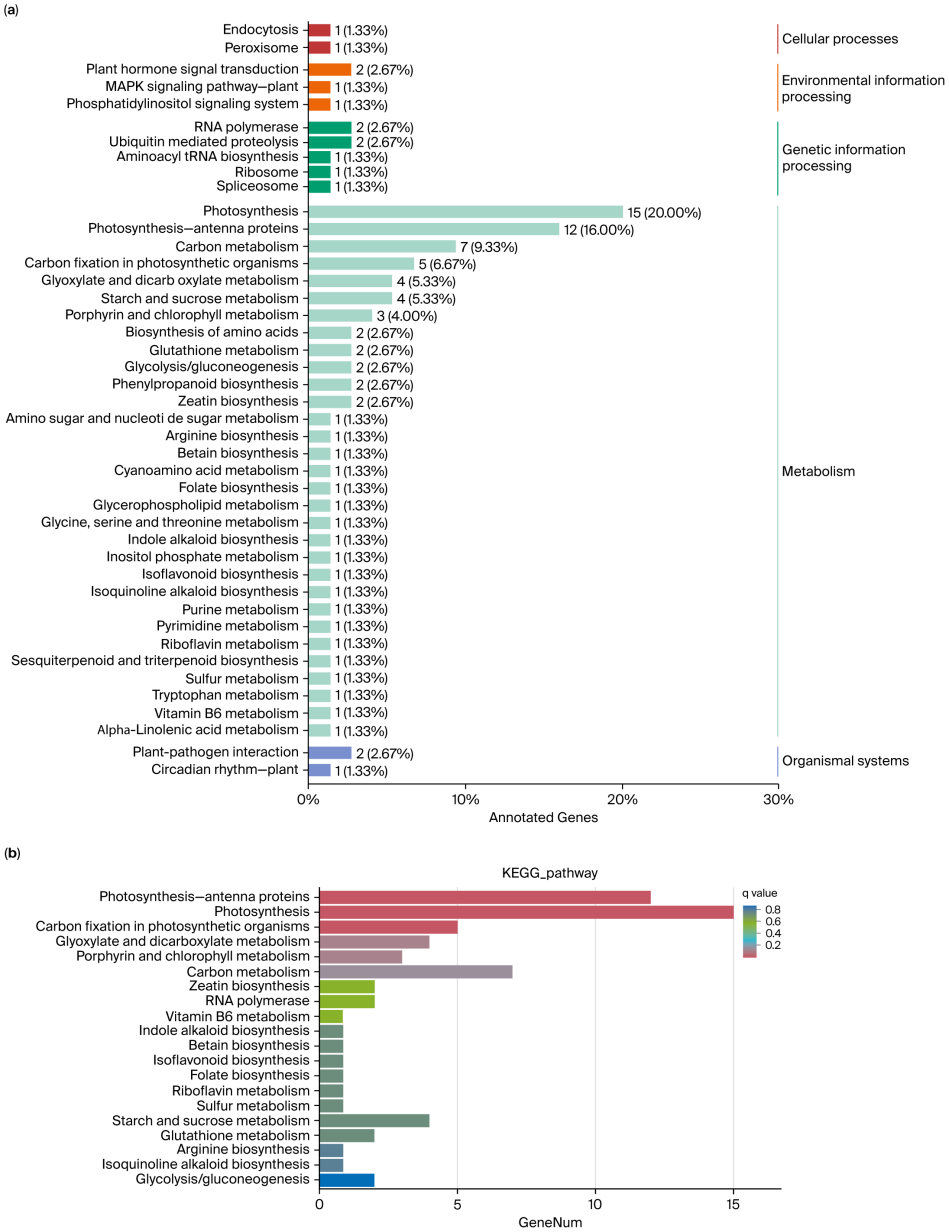
KEGG pathway enrichment analysis of DEGs in *Coix lacryma*-*jobi* under 19-day waterlogging treatment. (A) Functional categorization of DEG-associated pathways (*p* < 0.05). (B) Enrichment scores of significantly altered pathways (Fisher’s exact test, FDR ≤ 0.01).

The systemic characterization through Kyoto Encyclopedia of Genes and Genomes (KEGG) pathway annotation demonstrated significant enrichment of differentially expressed transcripts in core metabolic networks (Fig. 7, Figs. S4–S5, Table S6). Notably, photosynthetic machinery components constituted the most prominent functional cluster, with three distinct but interconnected pathways showing coordinated regulation: (i) photosystem II reaction center dynamics (ko00196), (ii) light-harvesting complex assembly (ko00195), and (iii) carbon fixation enzymatic cascades (ko00710). Complementary metabolic modules including glyoxylate–dicarboxylate interconversion (ko00630) and starch–sucrose partitioning systems (ko00500), exhibited concurrent activation. This multi-layered regulatory pattern suggests a concerted metabolic reprogramming strategy in response to photoperiodic stimuli, potentially facilitating enhanced photosynthetic efficiency and photoassimilate redistribution.

### Validation of differentially expressed genes *via* quantitative real-time PCR

**Figure 8:**
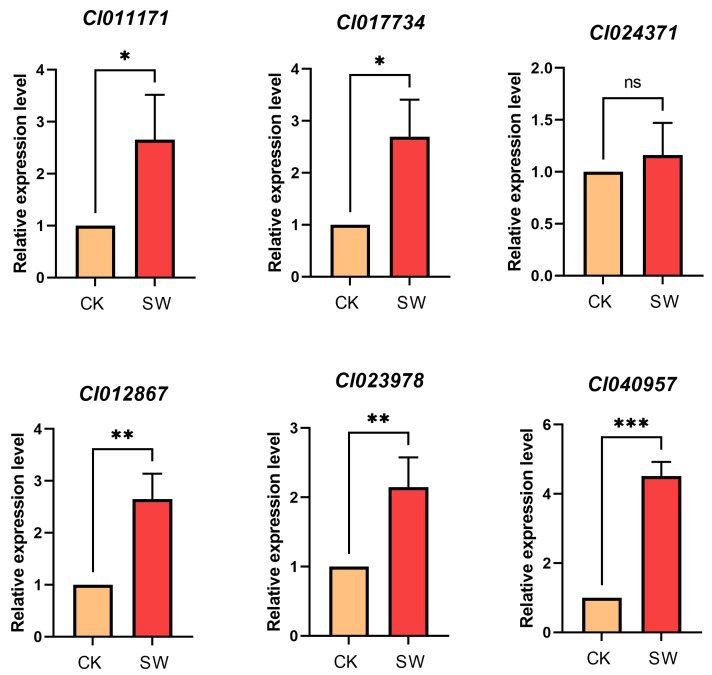
QRT-PCR validation of six key differentially expressed genes related to photosynthesis, carbohydrate transport and metabolism. Data are presented as mean ± SD (*n* = 3). 18S was used as an endogenous control. Asterisks denote significant differences based on Student’s *t*-test: *, *p* < 0.05; **, *p* < 0.01; ***, *p* < 0.001.

Functional validation of the prioritized candidate genes underpinning waterlogging adaptation was systematically conducted, with particular emphasis on two critical physiological mechanisms: photosynthetic homeostasis maintenance and carbohydrate allocation regulation. RNA-Seq revealed six core regulators, including three chloroplast-localized transcriptional regulators (*Cl011171*, *Cl017734*, and *Cl024371*) exhibiting significant differential expression patterns (fold change >5, *p* < 0.01) and three carbohydrate metabolism-related transporters and catalytic enzymes (*Cl012867*, *Cl023978*, and *Cl040957*) demonstrating environmental stress-induced transcriptional activation. The results of the qRT-PCR demonstrated consistent expression patterns consistent with the RNA-Seq data (Fig. 8, Table S1). Notably, the identified photosynthetic modulators are phylogenetically conserved in terrestrial plants. In contrast, the carbohydrate-associated genes displayed unique structural domains specific to flood-tolerant species, suggesting a functional importance in aquatic adaptation that warrants further investigation.

## Discussion

### Waterlogging stress constrains *Coix lacryma*-*jobi* development during the jointing phase

The physiological constraints imposed by waterlogged conditions primarily stem from oxygen deprivation in saturated soils, which induces hypoxic stress that disrupts plant metabolic processes (Sairam et al., 2009). During initial waterlogging exposure, plants exhibited sequential foliar responses, characterized by leaf reddening followed by yellowing, accelerated senescence, and diminished root activity accompanied by partial root blackening and decay. In advanced stages of waterlogging, accelerated root growth led to root-to-shoot ratio imbalance, a premature transition from vegetative to reproductive growth, leaf apoptosis, and severe impairment of aboveground biomass accumulation.

*Coix lacryma*-*jobi* demonstrates adaptive morphological features during growth, notably through the formation of a protective white powdery film on stem surfaces. This cuticular modification serves two functions: mitigating water loss under drought conditions and providing partial defense against waterlogging stress (Zhao, 2000). In our experimental observations during the jointing stage, while light and moderate waterlogging treatments showed measurable effects on plant height, culm diameter, and internode development; these parameters did not attain statistical significance compared to controls (Figs. 1–2, S6, Table S2). Leaf development remained largely unaffected at these stress levels. However, severe waterlogging induced significant suppression of vegetative growth parameters (*p* < 0.05). Root system architecture and dry matter accumulation exhibited particular sensitivity to waterlogging gradients, with progressive root blackening, decay, and biomass reduction correlating with increasing waterlogging severity (Figs. 3–4). Comparative analysis reveals that *Coix lacryma*-*jobi*’s growth response to waterlogging shares similarities with but also differs from major cereal crops. Like maize (*Zea mays*), *Coix* exhibits hypoxia-induced primary root growth arrest, but it also shows more pronounced adventitious root development than most maize varieties under comparable stress (Sairam et al., 2009). These findings align with established waterlogging responses observed in gramineous species. Comparative studies on maize (*Zea mays*) have documented hypoxia-induced primary root growth arrest and lateral root suppression, which are mitigated by adventitious root proliferation (Sairam et al., 2009). Under analogous conditions, cotton (*Gossypium hirsutum*) under analogous conditions showed a 30% height reduction and reproductive organ abscission (Zhang et al., 2021), while rice (*Oryza sativa*) displayed a similar decline in root oxidation capacity coupled with aerenchyma development to facilitate oxygen diffusion (Colmer & Voesenek, 2009). These response patterns conserved across species highlight the fundamental constraints on root meristematic activity and photoassimilate allocation imposed by hypoxic stress; *Coix lacryma*-*jobi* demonstrates an intermediate level of tolerance compared to aquatic and xeric-adapted species.

### Morphological plasticity of *Coix lacryma*-*jobi* in water-saturated environments: strategic adventitious root proliferation

Adventitious root systems emerging from hypocotyls and aerial organs constitute a critical survival response to soil hypoxia. As a principal morphological adaptation to prolonged waterlogging, these *de novo* roots functionally compensate for deteriorating primary roots through three synergistic mechanisms: (1) supplanting oxygen-deprived root segments, (2) optimizing oxygen absorption efficiency *via* structural reorganization, and (3) balancing carbohydrate metabolism through enhanced respiratory consumption. This root system remodeling not only ensures nutritional homeostasis but also reinforces mechanical stability during post-submergence recovery (Almeida, Vriezen & Straeten, 2003).

In our investigation, adventitious root proliferation was dependent on the waterlogging intensity, with a particularly pronounced manifestation in the moderate waterlogging treatment group. Statistical analysis revealed significant differences in adventitious root development between the control group and both the moderate and severe waterlogging treatments, though not in the light waterlogging group (Fig. 3). This intensity-dependent response aligns with prior observations reported in Poaceae species. The adventitious root development pattern in *Coix lacryma*-*jobi* appears intermediate between the extensive systems formed by rice and the more limited development in most maize genotypes. While rice cultivars subjected to partial submergence exhibited 200–300% increases in nodal adventitious roots compared to aerated controls (Sauter, 2005), *Coix* showed a more moderate but still significant increase. Similarly, *Zea mays* ssp. *huehuetenangensis* seedlings develop extensive adventitious root networks when exposed to transient flooding (Mano et al., 2005), but commercial maize hybrids generally show poorer adventitious root development. Comparative studies on barley further corroborate this pattern, with tolerant genotypes producing 58% more adventitious roots than sensitive counterparts after three weeks of submergence (Luan et al., 2018), a response magnitude similar to what we observed in *Coix*. These convergent evolutionary responses highlight *Coix lacryma*-*jobi*’s sophisticated strategy for hypoxia mitigation through controlled root system plasticity, indicating that it has moderate to good waterlogging tolerance compared to other cereals.

### Metabolic adaptation strategies in *Coix lacryma*-*jobi*: carbon allocation and photosynthetic responses to waterlogging

Under hypoxia, plants activate metabolic adaptations to sustain energy production independent of mitochondrial oxidative phosphorylation (Braun, 2020). Pyruvate acts as a pivotal node connecting cytoplasmic glycolysis to mitochondrial respiration. Following glycolytic processing, pyruvate enters the mitochondria and undergoes oxidation *via* the tricarboxylic acid (TCA) cycle, yielding organic acids and NADH to fuel ATP synthesis (Braun, 2020).

Integrated RNA-Seq analysis combined with COG/GO/KEGG functional annotation revealed extensive reprogramming of carbohydrate homeostasis pathways in *Coix lacryma*-*jobi* under waterlogged soil conditions (Figs. 5–7, Figs. S1–S5, Table S1). The key differentially expressed genes (DEGs) were validated using qRT-PCR. Three central metabolic genes showed conspicuous upregulation in both datasets (Fig. 8; Table S1): *Cl040957* encodes ribulose bisphosphate carboxylase (Rubisco), the Calvin cycle enzyme initiating inorganic carbon fixation and linking abiotic carbon assimilation to biosynthesis; *Cl023978* encodes glyceraldehyde-3-phosphate dehydrogenase A (GAPDH A), catalyzing glyceraldehyde-3-phosphate oxidation and NADH generation during glycolysis; and *Cl012867* encodes fructose-1,6-bisphosphatase, a chloroplastic/cytosolic enzyme coordinating the Calvin cycle, gluconeogenesis, and sucrose synthesis within carbon metabolic networks. The metabolic reprogramming observed in *Coix lacryma*-*jobi* shows both convergence and divergence with other cereals in this regard. Similar to rice, *Coix* maintains glycolytic flux and fermentative pathways under hypoxia, but unlike the ethanol-dominated fermentation in rice, *Coix* appears to utilize a more diverse set of alternative pathways, including lactate and alanine production (Elaine et al., 2018). Compared to maize, which shows rapid depletion of carbohydrate reserves under waterlogging, *Coix* demonstrates better maintenance of soluble sugars, resembling the pattern observed in waterlogging-tolerant barley genotypes (Herzog et al., 2016). The observed upregulation of these genes in root tissues likely reflects systemic signaling and metabolic integration between shoot and root organs. Research has demonstrated that light-signaling components, including HY5 protein, can translocate from shoots to roots to modulate carbon-nitrogen balance and auxin distribution, thereby influencing root architecture (Gao, Chao & Lin, 2008). This shoot-to-root communication may explain the expression of photosynthetic genes in root tissues as part of a coordinated whole-plant response to stress. This concerted upregulation implies enhanced TCA cycle activity and glycolytic flux in *Coix lacryma*-*jobi* to counteract energy deficits under hypoxia, aligning with prior reports of waterlogging-induced Rubisco elevation in cotton (Kuai et al., 2014).

Photosynthesis underpins autotrophic growth in higher plants, influencing both ecological resilience and agricultural productivity. Waterlogging stress accelerates chlorophyll degradation in leaves (Ashraf & Arfan, 2005), promoting premature senescence and leaf shedding while diminishing photosynthetic capacity. Tolerant species, however, maintain photosynthetic integrity through structural stability and elevated photosynthetic rates under hypoxia (Tong et al., 2021). Genetic evidence supports the notion that introducing the *Sub1A* gene from tolerant rice lines enhances waterlogging tolerance *via ADH1* upregulation (Xu et al., 2006).

Notably, although our RNA-Seq data were generated exclusively from root tissues, we observed significant upregulation of several genes traditionally associated with photosynthetic processes. This phenomenon can be explained by the multifaceted roles of these gene products beyond their canonical functions in leaves. For instance, components of the photosynthetic electron transport chain, such as those encoded by PSI-related genes, are also utilized in non-photosynthetic plastids for alternative electron transport processes that are crucial for stress adaptation (Shikanai & Yamamoto, 2017). Our RNA-Seq data further indicated waterlogging-induced modifications in photosynthetic pathways (Figs. 6–7, S1–S5), which were corroborated using qRT-PCR. Three critical photosynthetic genes showed marked upregulation: *Cl011171* encodes the PSI reaction center subunit psaK, which stabilizes PSI architecture, optimizes light harvesting and electron transport, and bolsters stress resilience (Jensen et al., 2000); *Cl017734* encodes the NDH subcomplex B4 subunit, mediating cyclic electron flow to (i) balance ATP/NADPH ratios for the Calvin cycle, (ii) provide photoprotection *via* PSI redox regulation, and (iii) fortify abiotic stress tolerance (Shikanai et al., 1998; Yuri et al., 2002), and *Cl024371* encodes a core PSI subunit that coordinates light-energy conversion and electron transfer through multi-subunit interactions (Amunts & Nelson, 2009; Nelson & Ben-Shem, 2010).

The expression patterns of photosynthetic genes in *Coix lacryma*-*jobi* roots subjected to waterlogging reveal interesting differences relative to other cereals. While rice maintains photosynthetic capacity through the QUICK AND SLOW RELATIONSHIP (QSR) mechanism involving *Sub1A* gene regulation, and maize typically shows rapid photosynthetic decline, *Coix* demonstrates an intermediate strategy that combines elements of both tolerance and avoidance (Panda & Barik, 2021). The upregulation of PSI-related genes in *Coix* roots resembles the response observed in waterlogging-tolerant barley genotypes, indicating conserved mechanisms for managing redox homeostasis under hypoxia across these species. The expression of these genes in root tissues likely reflects their involvement in non-photosynthetic functions essential for hypoxic stress responses. In heterotrophic organs such as roots, plastidial electron transport chains contribute to maintaining redox homeostasis and generating ATP under energy-limiting conditions (Shikanai & Yamamoto, 2017; Yamamoto & Shikanai, 2019). Specifically, the NDH-mediated cyclic electron flow around PSI helps alleviate over-reduction in the electron transport chain and mitigates reactive oxygen species (ROS) generation under stress (Yamamoto & Shikanai, 2019). Collectively, the upregulation of these genes in *Coix lacryma-jobi* roots suggests a strategic enhancement of plastidial electron transport components to mitigate waterlogging-induced energy crisis and oxidative stress, rather than representing direct involvement in photosynthetic carbon assimilation.

### Future directions

This study clarified the adaptive responses of *Coix lacryma*-*jobi* to waterlogging stress through controlled irrigation trials and RNA-Seq profiling. However, limitations in the experimental data warrant discussion. Although waterlogging commonly impairs photosynthetic efficiency, essential photosynthetic metrics were omitted in this study. Follow-up studies may utilize gas-exchange monitoring systems to compare the photosynthetic rates between stressed and control plants or, alternatively, assess chlorophyll content as a proxy for photosynthetic function.

Waterlogging significantly affects root development by depleting soil oxygen (O_2_) and restricting O_2_ diffusion within tissues, thereby disrupting metabolic processes and growth (Guo et al., 2020; Voesenek & Bailey-Serres, 2013). Future experiments should quantify root architectural parameters (*e.g.*, length, surface area) to address this gap.

Notably, the prompt activation of anaerobic respiration (*via* ethanol/lactate fermentation) in waterlogged plants enhances glycolytic flux to sustain energy supply under hypoxia (Colmer & Voesenek, 2009). To verify the physiological consequences of waterlogging in *Coix lacryma*-*jobi*, key metabolites require quantification, including the activities of pyruvate decarboxylase (PDC), alcohol dehydrogenase (ADH), malondialdehyde (MDA), catalase (CAT), and superoxide dismutase (SOD).

The statistical power of 0.72, resulting from a moderate sample size of three biological replicates and the absence of technical replicates, is a limitation of this study. This constraint may have reduced our sensitivity with respect to detecting differentially expressed genes (DEGs), particularly those with subtle expression changes or high intrinsic variability. We will incorporate both a larger sample size and technical replicates in future experimental designs to achieve more comprehensive and robust gene discovery.

The proliferation of adventitious roots in stressed plants suggests a morphological adaptation that is potentially modulated by abscisic acid (ABA). As a critical regulator of waterlogging responses, ABA induces stomatal closure, suppresses transpiration, and inhibits lateral root formation (Yoon-Ha et al., 2015). Our RNA sequencing data identified differentially expressed genes, including *Cl001821* and *Cl028721*, which are potentially involved in ABA biosynthesis. Analysis of cis-regulatory elements in the promoters of *Cl001821* and *Cl028721* revealed the presence of both the ABA-responsive element (ABRE) and the anaerobic stress-responsive element (ARE). Furthermore, *Cl001821* contains a GC-motif, indicating a potential role in hypoxia-specific induction. These promoter features support the need for expression profiling of these candidate genes to further elucidate their roles in the ABA signaling pathway.

Stress-responsive transcription factors uncovered in the RNA-Seq analysis—particularly *NewGene_4125*, which encodes a a zinc finger protein—may coordinate waterlogging adaptation. These findings delineate promising avenues for refining experimental frameworks. Subsequent research should integrate physiological markers with molecular datasets to comprehensively analyze the regulatory networks underpinning waterlogging tolerance in *Coix lacryma*-*jobi*.

## Conclusions

Our findings demonstrate that *Coix lacryma*-*jobi* employs a three-tiered adaptive strategy when faced with waterlogging stress: (1) morphological plasticity manifested as a 76.6% increase in adventitious root proliferation and a 14.4–21.8% reduction in critical growth parameters (culm diameter, plant height, and dry biomass); (2) RNA-Seq analysis identified 207 differentially expressed genes (DEGs), with functional enrichment revealing predominant engagement in photosynthetic electron transport (KEGG:0015979), starch/sucrose metabolism (KEGG:00500), and ethylene signaling pathways (KEGG:04075); and (3) six core regulatory genes (*Cl040957*, *Cl023978*, *Cl017734*, *Cl011171*, *Cl024371*, and *Cl012867*) were validated through qRT-PCR, showing induction in photosynthetic apparatus components and carbohydrate transporters. Together, these coordinated responses may represent a physiological trade-off between growth restraint and metabolic maintenance, whereby the temporary suppression of vegetative growth is counterbalanced by enhanced root aerenchyma formation and more efficient carbon allocation. The identified DEGs constitute promising candidates for further investigation into molecular targets for marker-assisted breeding of flood-tolerant *Coix* varieties.

## Supplemental Information

10.7717/peerj.20731/supp-1 Supplemental Information 1 Raw data

10.7717/peerj.20731/supp-2 Supplemental Information 2 Gene Ontology biological process enrichment analysis of differentially expressed genes between control (CK) and treatment (T) groups (bar plot)

10.7717/peerj.20731/supp-3 Supplemental Information 3 Gene Ontology cellular component enrichment analysis of differentially expressed genes between control (CK) and treatment (T) groups (bar plot)

10.7717/peerj.20731/supp-4 Supplemental Information 4 Gene Ontology molecular function enrichment analysis of differentially expressed genes between control (CK) and treatment (T) groups (bar plot)

10.7717/peerj.20731/supp-5 Supplemental Information 5 KEGG pathway enrichment analysis of differentially expressed genes between control (CK) and treatment (T) groups (dot plot)

10.7717/peerj.20731/supp-6 Supplemental Information 6 Gene-concept network visualization of KEGG pathway enrichment results for control (CK) vs. treatment (T) comparison (cnet plot)

10.7717/peerj.20731/supp-7 Supplemental Information 7 Effects of waterlogging on leaf development in *Coix lacryma*-*jobi* during the jointing stage

10.7717/peerj.20731/supp-8 Supplemental Information 8 Primers for qRT-PCR validation of the differentially expressed genes

10.7717/peerj.20731/supp-9 Supplemental Information 9 Internode lengths per plant of *Coix lacryma*-*jobi* under 19 -day waterlogging treatments treatment and non-waterlogged control conditions

10.7717/peerj.20731/supp-10 Supplemental Information 10 DEGs in *Coix lacryma*-*jobi* under 19-day waterlogging treatment between control and waterlogging treatment groups

10.7717/peerj.20731/supp-11 Supplemental Information 11 COG annotation of the DEGs in *Coix lacryma*-*jobi* under 19-day waterlogging treatment between control and waterlogging treatment groups

10.7717/peerj.20731/supp-12 Supplemental Information 12 GO enrichment of the DEGs in *Coix lacryma*-*jobi* under 19-day waterlogging between control and waterlogging treatment groups

10.7717/peerj.20731/supp-13 Supplemental Information 13 KEGG enrichment of the DEGs in *Coix lacryma*-*jobi* under 19-day waterlogging treatment between control and waterlogging treatment groups

10.7717/peerj.20731/supp-14 Supplemental Information 14 MIQE specification

10.7717/peerj.20731/supp-15 Supplemental Information 15 MIQE checklist

## Additional Information and Declarations

### Data Availability

The following information was supplied regarding data availability: The raw data are available in the Supplemental Files.
