# Supplementary figures and images for: Morphological characterization and RNA sequencing reveal adaptive strategies of Coix lacryma-jobi L. under waterlogging stress during the jointing stage
Source: PeerJ. 2026 Feb 5;14:e20731. doi: 10.7717/peerj.20731 (PMC12883155; doi:10.7717/peerj.20731)

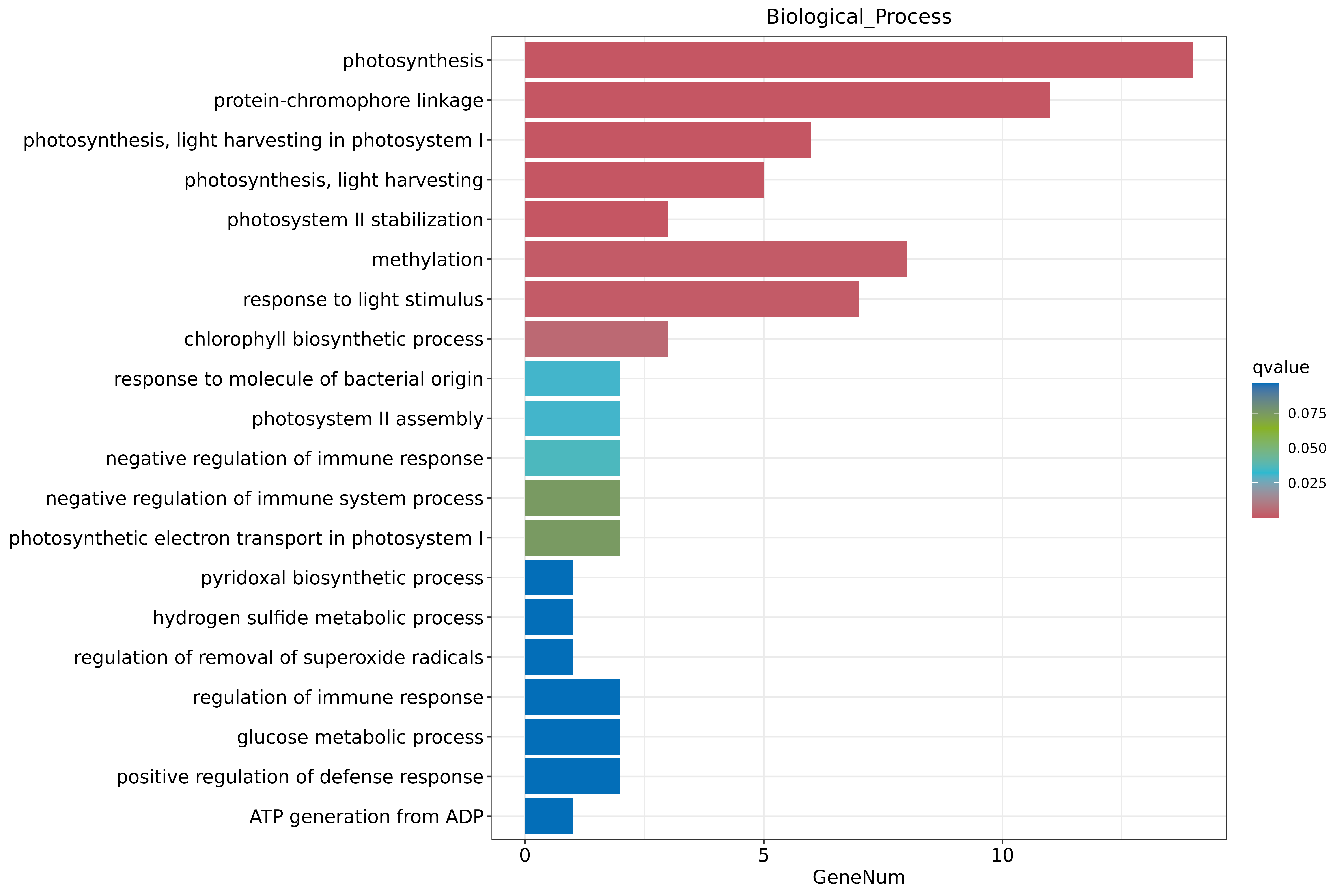

Supplement: Supplemental Information 2 [file peerj-14-20731-s002.png]

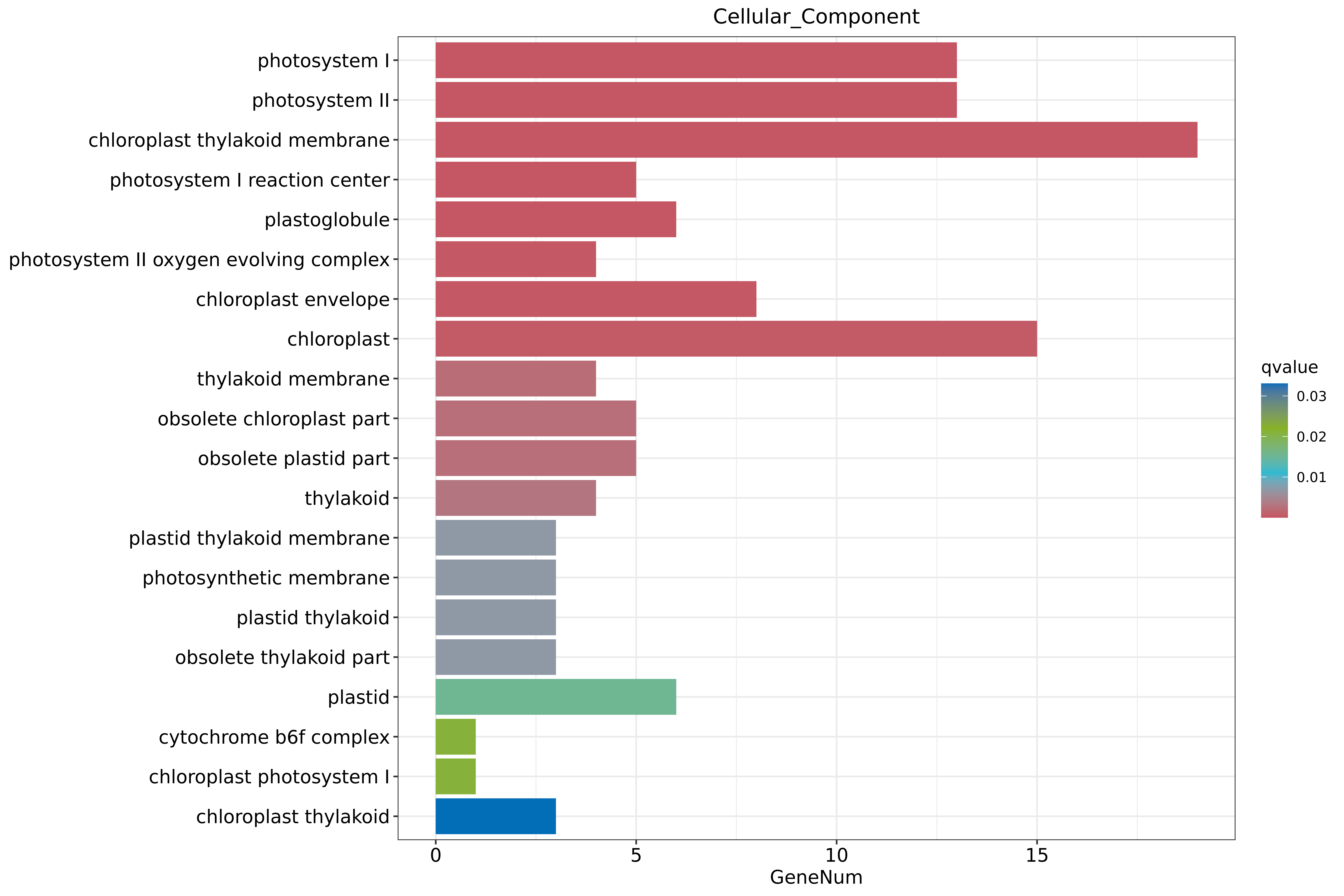

Supplement: Supplemental Information 3 [file peerj-14-20731-s003.png]

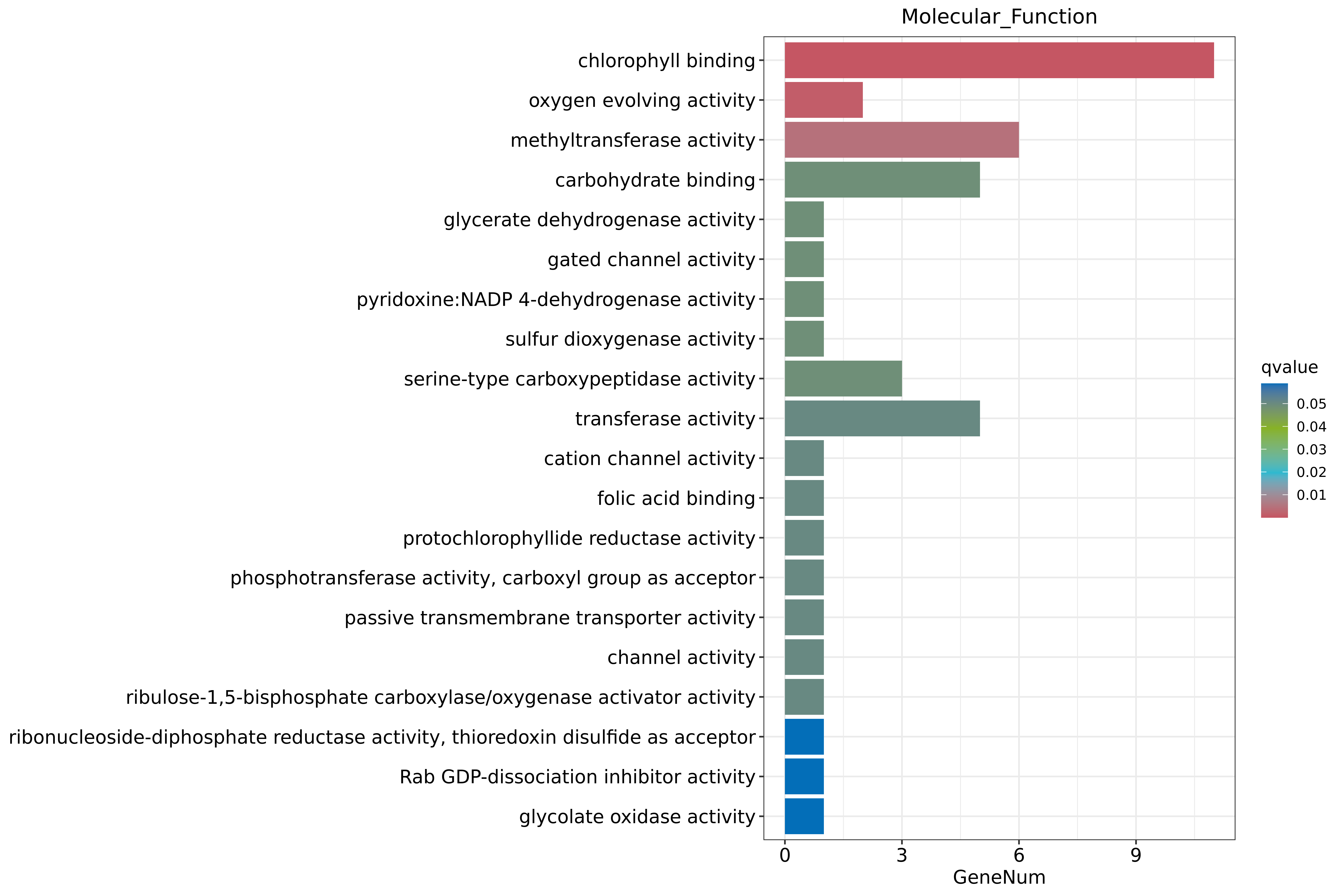

Supplement: Supplemental Information 4 [file peerj-14-20731-s004.png]

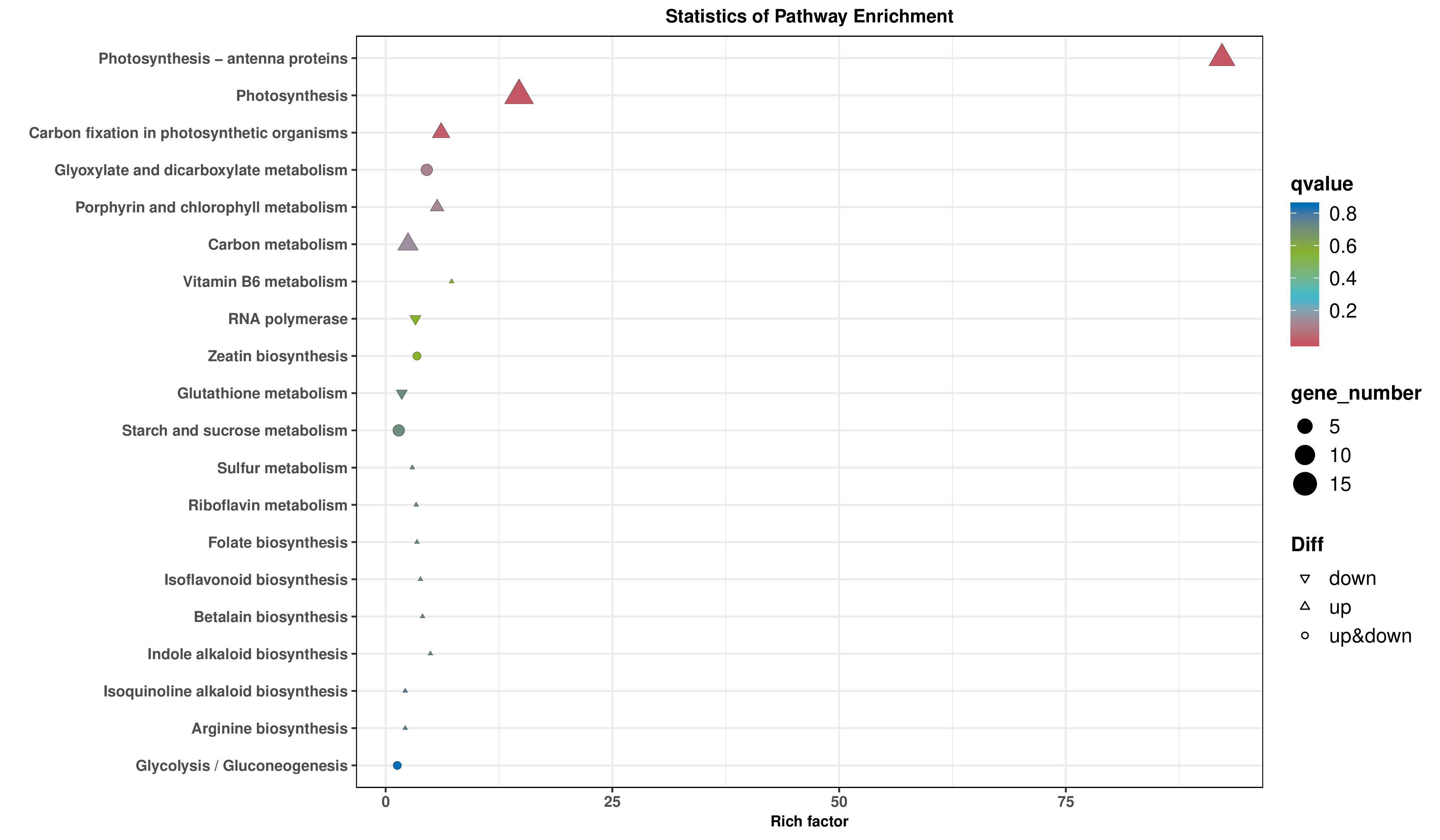

Supplement: Supplemental Information 5 [file peerj-14-20731-s005.png]

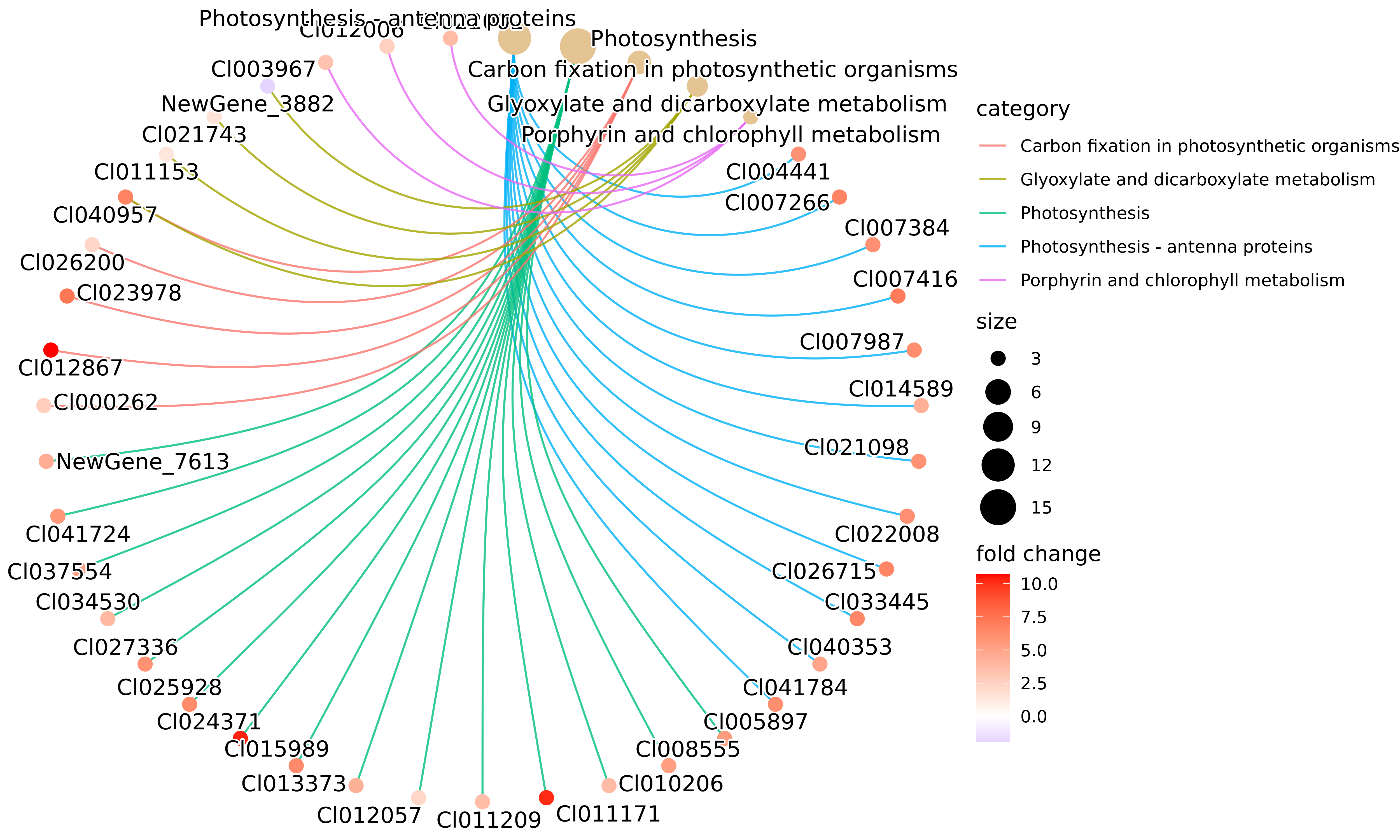

Supplement: Supplemental Information 6 [file peerj-14-20731-s006.png]

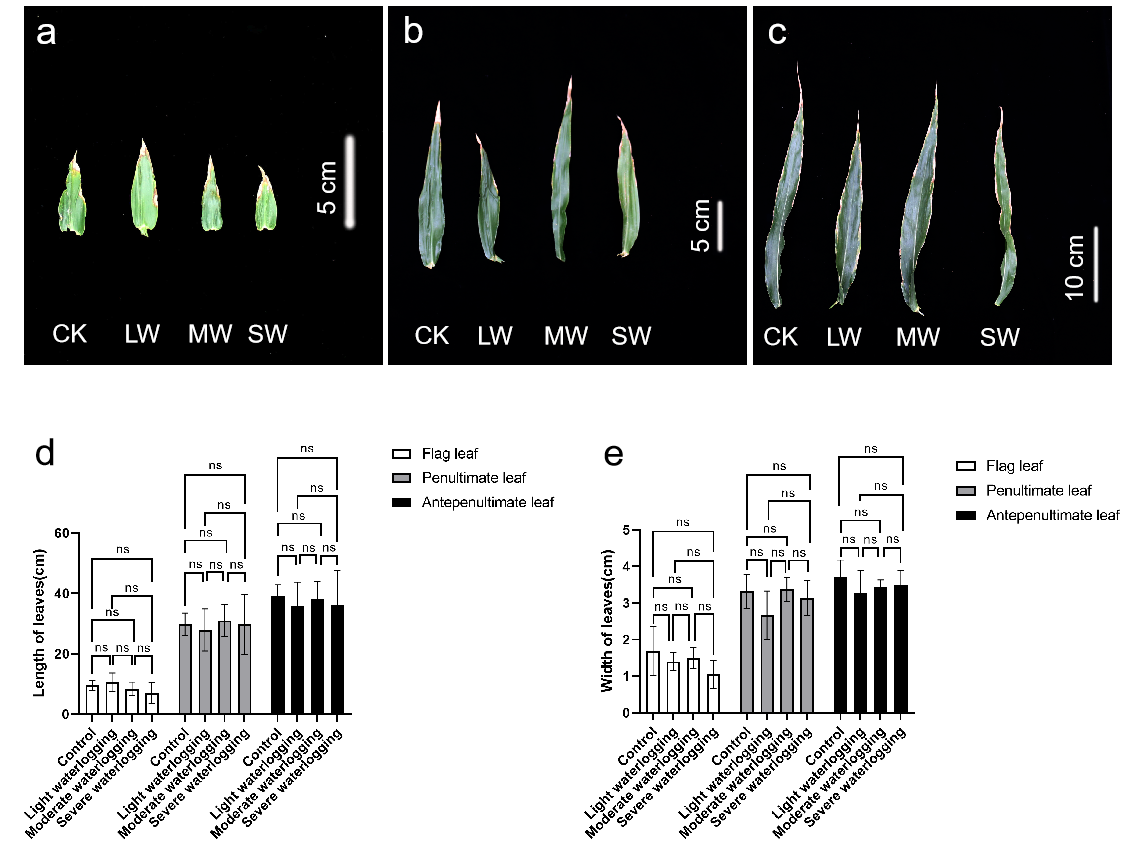

Supplement: Supplemental Information 7 [file peerj-14-20731-s007.tif]
