# Supplementary material for: Morphological characterization and RNA sequencing reveal adaptive strategies of Coix lacryma-jobi L. under waterlogging stress during the jointing stage
Source: PeerJ. 2026 Feb 5;14:e20731. doi: 10.7717/peerj.20731 (PMC12883155; doi:10.7717/peerj.20731)
Supplement: Supplemental Information 14 [file peerj-14-20731-s014.docx]

MIQE specification file

This study utilized root specimens from *Coix lacryma*-*jobi* L., comprising two groups: waterlogged treatment and control, each with three biological replicates. Roots from fresh plants of both groups were sectioned and immediately flash-frozen at -80°C.

Reagents were commercially sourced from Tiangen Biotech (Beijing) Co., Ltd. and GenStar Biosolutions Co., Ltd.

1. **Experimental instruments**
2. BSA124S analytical balance of Sartorius AG
3. 1730R high-speed refrigerated centrifuge of Gene Tech (Wuhan) Co., Ltd.
4. DYY-7C electrophoresis of Liuyi Biotech (beijing) Co.,Ltd.
5. NanoDrop 2000 microspectrophotometer of Thermo Fisher Scientific
6. SimpliAmp™ thermal cycler of Thermo Fisher Scientific
7. Bio-Rad CFX connect real-time PCR platform of Bio-Rad Laboratories

**2. Total RNA isolation:** Experimental materials were removed from centrifuge tubes, flash-frozen in liquid nitrogen, ground, and processed for total RNA extraction using the Tengen kit (DP432; components in Table 1) according to the manufacturer's protocol. This kit facilitates rapid, high-purity RNA isolation from multiple diverse plant samples simultaneously, yielding RNA free of protein contamination suitable for downstream applications including RT-PCR, Real Time RT-PCR, microarray analysis, Northern Blot, Dot Blot, PolyA screening, in vitro translation, RNase protection analysis, and molecular cloning.

RNA purity and structural integrity were evaluated by spectrophotometry (NanoDrop 2000; A260/A280 and A260/230 ratios), with confirmation via electrophoresis on 1% agarose gels (1.2% gel concentration, 0.5x TBE buffer, 150 V, 15 min). As cellular RNA is predominantly (70-80%) rRNA, distinct rRNA bands should be visible under UV. Expected bands correspond to ~5 kb (28S) and ~2 kb (18S) rRNA; plant leaf RNA may show additional bands due to chloroplast rRNA. Crucially, the largest rRNA band intensity should be 1.5-2.0 times that of the next largest band. Ratios below this range, diffuse bands, or band disappearance indicate degradation. Extracted RNA was measured for concentration and stored at -80 °C.

Table 1. RNA Extraction Kit Components

| Serial number | Reagent name | Specification |
| --- | --- | --- |
| 1 | Bufter RL | 30 mL |
| 2 | Bufer RW1 | 40 mL |
| 3 | Buffer RW | 12 mL |
| 4 | RNase-Free ddH2O | 15 mL |
| 5 | RNase-Free Columns CR3set | 50 sets |
| 6 | RNaseFreeColumnsCS set | 50 sets |
| 7 | RNaseFree Centrifuge Tubes (1.5ml) | 50 |
| 8 | RNase-Free DNasel(1500U) | 1 |
| 9 | BufferRDD (DNADigest Bulfer) | 4 mL |
| 10 | RNase-Free ddH_2_O | 1 mL |

**3. Synthesis of cDNA first strand (reverse transcription):** Using 800 ng of total RNA per sample as template, reverse transcription was performed in a 20 µL reaction system containing 4 µL of GenStar Biosolutions Co., Ltd.'s 5× StarScript Pro All-in-one RT Mix with gDNA Remover (A240-10; Table 2) and nuclease-free water (DEPC-treated). This pre-mixed kit enables simultaneous genomic DNA removal and cDNA synthesis within a single 5-minute step, minimizing contamination risk and RNA degradation. It features highly efficient StarScript Pro reverse transcriptase and an optimized buffer containing Random Primer and Oligo18 (dT), eliminating the need for separate primers. The buffer ensures consistent reverse transcription efficiency across varying RNA concentrations, particularly for short-stranded cDNA synthesis. The reaction program was: 37°C for 2 min, 50°C for 15 min, and 85°C for 2 min. Resulting cDNA, compatible with both dye-based and probe-based qPCR for subsequent gene expression analysis, was immediately placed on ice or stored at -20°C.

Table 2. Components of the Gene star cDNA First Strand Synthesis Kit

| Component item number | Reagent name | Specification |
| --- | --- | --- |
| ZA240-101 | StarScript Pro All-in-one RT Mix | 100 µl |
| ZA240-102 | 5×StarScript Pro All-in-one RT Buffer | 400 µl |
| ZA240-103 | No RT Control Mix | 10 µl |
| ZA220-101 | Nuclease-free Water (DEPC-treated) | 1.5 ml |

**4. Primer Design:** Gene sequences were input into NCBI Primer-BLAST with parameters set to: product length 80-300 bp; Tm 55-65 °C. Candidate primer pairs exhibiting uniform GC distribution and matching Tm values were selected. Using Primer Premier 5.0, potential primer dimers and hairpin structures were assessed, and 3′ end bases were adjusted to enhance specificity. Primer sequence uniqueness and absence of non-target binding sites were confirmed via NCBI BLAST against the transcriptome. Synthesized primers (Table 3) were first validated by PCR and specificity testing via gel electrophoresis before subsequent experiments.

Table 3. Primers for qRT-PCR validation of the differentially expressed genes.

| Primer ID | Primer Sequence | Primer Tm | Final concentration (μM) | Amplification size (bp) |
| --- | --- | --- | --- | --- |
| Cl040957_1_F | CTACGACGGCCGCTACTG | 59.976 | 10 | 100 |
| Cl040957_1_R | GTAGGCGGCGATGGTCTC | 59.972 | 10 |  |
| Cl023978_1_F | CAACCACGACGAGCCCAT | 60.047 | 10 | 114 |
| Cl023978_1_R | ATCCCGTTGAGCTTGCCC | 60.045 | 10 |  |
| Cl017734_1_F | GCTGAACGACCATGGCCT | 60.046 | 10 | 149 |
| Cl017734_1_R | CCCGTGGTCATGCAACCT | 59.966 | 10 |  |
| Cl011171_1_F | CGCGCTCGACCAGATCAT | 59.973 | 10 | 117 |
| Cl011171_1_R | TACATAGGCACCGCGTCG | 59.59 | 10 |  |
| Cl024371_1_F | CGCACCACAAAACCAGGC | 59.973 | 10 | 111 |
| Cl024371_1_R | TCGGTCGGTCGAGTCTGT | 59.968 | 10 |  |
| Cl012867_1_F | GACGTGCACCACTCCCTC | 60.048 | 10 | 110 |
| Cl012867_1_R | GTGCGCTGCTAACGTTGG | 59.827 | 10 |  |
| 18S-F | GGATCGGAGTAATGATTAACAGGG | 59.867 | 10 | 138 |
| 18S-R | CGTTTATGGTTGAGACTAGGACGG | 59.887 | 10 |  |

**5. Quantitative Real-Time PCR (qPCR):** cDNA synthesized by reverse transcription was diluted 10-fold and analyzed using SYBR Green chemistry (A301-05; Table 3) on a Bio-Rad qPCR instrument.

The reaction employed a 2× premix for SYBR Green I-based real-time fluorescence quantitative PCR. This optimized formulation contains GenStar Fast HSTaq DNA Polymerase, SYBR Green I, dNTPs, Mg²⁺, reaction buffer, and stabilizer, and is suitable for detecting both genomic DNA and cDNA targets.

Table 3. Components of the 2×RealStar Fast SYBR qPCR Mix

| Reagent name | Specification |
| --- | --- |
| 2×RealStar Fast SYBR qPCR Mix | 1.1 ml×5 |
| High/Low ROX Reference Dye | Optional according to the instrument model |

Key Features & Mechanism:

1. Hot-Start Taq: An anti-Taq monoclonal antibody binds to the polymerase at low temperatures, inhibiting its activity and preventing non-specific amplification (e.g., from primer-template mishybridization or primer dimer formation). This antibody is irreversibly inactivated during the initial PCR denaturation step.
2. Optimized SYBR Green I: Enhanced concentrations ensure strong fluorescence upon binding double-stranded DNA, with minimal background signal from unbound dye. This allows fluorescence measurement during annealing/extension, ensuring signal increase directly correlates with PCR product accumulation.
3. Performance: The system offers speed, simplicity, high sensitivity, high specificity, and good stability, minimizing human error, saving time, and reducing contamination risk.

Reaction Setup: Prepare a 1× working concentration by combining:

1. 5 μL 2× SYBR Green Premix
2. 1 μL cDNA template
3. 0.5 μL Forward Primer
4. 0.5 μL Reverse Primer
5. 3 μL ddH₂O

(Optional: Add ROX Reference Dye if required by the instrument for well-to-well signal correction).

Thermal Cycling Protocol:

1. 50°C for 2 min
2. 94°C for 10 min (initial denaturation/antibody inactivation)
3. 40 cycles of:
4. 94°C for 15 s (denaturation)
5. 60°C for 30 s (annealing)
6. 72°C for 30 s (extension)

Melt curve analysis

After the reaction completes, results are automatically saved. Baseline parameters (Start: 3-15, End: 5-20) and Threshold are then adjusted through image analysis to flatten the negative control's amplification curve below the threshold line. Users may customize these baseline settings as needed. Finally, click Analyze to record the Ct value.

**6. qPCR Data Analysis:** Gene expression levels were quantified using the 2^-ΔΔCT^ method, with 18s rRNA serving as the endogenous control. Statistical significance of the results was calculated and visualized using GraphPad Prism software.
